# Virtual reality solution to promote adapted physical activity in older adults: outcomes from VR2Care project exploratory study

**DOI:** 10.3389/fpubh.2025.1584406

**Published:** 2025-05-13

**Authors:** Vincenzo De Luca, Malak Qbilat, Alessandra Cuomo, Antonio Bianco, Francesca Cesaroni, Chiara Lanari, Ad van Berlo, Telma Mota, Lucia Pannese, Michael Brandstötter, Matthieu Arendse, Vania Mota, Willeke van Staalduinen, Hugo Paredes, Guido Iaccarino, Maddalena Illario

**Affiliations:** ^1^Department of Public Health, Federico II University of Naples, Naples, Italy; ^2^Institute for Systems and Computer Engineering, Technology and Science (INESC TEC), Porto, Portugal; ^3^Department of Clinical Medicine and Surgery, Federico II University of Naples, Naples, Italy; ^4^Cooperativa Sociale COOSS MARCHE ONLUS scpa, Ancona, Italy; ^5^Smart Homes, Eindhoven, Netherlands; ^6^Altice Labs, Aveiro, Portugal; ^7^imaginary Srl, Milan, Italy; ^8^cogvis software und consulting GmbH, Wien, Austria; ^9^Tante Louise, Bergen op Zoom, Netherlands; ^10^Venerável Ordem Terceira de São Francisco do Porto, Porto, Portugal; ^11^Academy on Age-Friendly Environments in Europe (Afedemy), Gouda, Netherlands; ^12^Interdepartmental Research Center for Hypertension and related Conditions, University of Naples Federico II, Naples, Italy

**Keywords:** physical activity, virtual reality, older adults, gaming, digital health, telemonitoring, health promotion

## Abstract

**Background:**

Insufficient physical activity is one of the leading risk factors for death worldwide. Regular exercise can improve physical performance and quality of life, reduce the risks of falls and depressive symptoms, and reduce the likelihood of cognitive decline in older adults. Virtual reality (VR) and serious games (SG) are promising tools to improve physical and cognitive functioning. As part of the VR2Care project activities, four pilot sites explored the capabilities of the VR environment in a remote psychomotor training with SG and a hybrid approach with local groups of older adults performing physical activity.

**Objective:**

The present study aimed to explore and measure the impact on older adults’ quality of life and physical activity of using VR2Care solution and the level of usability, satisfaction and acceptance.

**Methods:**

The study is a mixed method study, using qualitative and quantitative surveys to evaluate quality of life and physical activity of older users, and usability, satisfaction and acceptance of the solution. The data collection is a mix of investigator site data entry and users’ self-reported data through the solutions or through online and paper-based means. Data were collected at baseline and after a follow-up of 6 weeks. Data are expressed as mean ± standard deviation (SD) unless otherwise stated. Within the group, baseline to end of observation differences were assessed by paired sample t-test. A *p* = 0.05 was considered significant.

**Results:**

No significant improvements in quality of life and physical activity were found. Little improvement, although not significant, in physical activity was found, comparing the Total MET average value of users who participated in phase I and II, therefore using SmartAL and Rehability. Little improvement, although not significant, in physical activity applies in ≥76 population. Users’ feedback on usability, satisfaction and acceptance of VR2Care is generally positive. VR2Care was appreciated mostly for its usefulness in managing physical activity and the capacity to influence the consistency of attending physical activity sessions as prescribed by doctors.

**Conclusion:**

Our results suggest that randomized controlled trial will be needed to assess correlations between specific features of the solution and health outcomes.

## 1 Introduction

The economic burden of population aging on health systems is the major challenge driving the urge to find innovative, cost-effective technological solutions (1). Such challenges have been further complicated by the emergence of the COVID-19 pandemic, when multilayered systemic interdependencies spread the effects of the pandemic across social, technological, economic, and healthcare dimensions (2). The effects of Covid-19 pandemics occurred mainly among vulnerable subgroups of the population (3), with an increased risk of adverse health outcomes for the older adult population. In this scenario, there was a strong push for innovative ways of communication and organizational approaches in healthcare, while the development of digital technologies accelerated, redefining economic, learning, professional and social systems, and environments (1). These approaches should be in line with the paradigmatic shift from reactive disease management toward early diagnosis and risk stratification, health promotion and prevention, and self-management (4). The demographic changes taking place globally have an increasing impact on the onset of multiple dysfunctions and the consequent loss of independence due to the aging of the population (5). There is an increasing need for effective and sustainable interventions that can provide opportunities for physical, psychological, and cognitive stimulation, as well as motivation for older adults (6). In general, physical activity has significant health benefits and contributes to preventing and managing non-communicable diseases (7). Globally, 31% of population is not active enough (8) and insufficient physical activity is one of the leading risk factors for death worldwide (9). Regular exercise can improve physical performance and quality of life, reduce the risks of falls and depressive symptoms, and reduce the likelihood of cognitive decline in older adults (10, 11). The prevalence of disability is expected to grow, due to population aging (12). Falls are the leading cause of injuries in older adults (13), and approximately one third of adults 65 years of age or older fall once a year (14). Access to rehabilitation can be problematic because rehabilitation providers are unavailable or in very small numbers, especially in low-income countries (15), and mainly concentrated in urban locations (16). A promising tool to improve physical and cognitive functioning is virtual reality (VR), a user-computer interface involving real-time stimulation and interactions of an embedded subject through multiple sensorial channels, based on a synthetic environment in which the subject feels his presence (17). Virtual reality allows to combine cognitive and physical exercises, improving cognitive performance more than either type of training alone (18). Physical exercise with embodied youth avatars affects perceived exertion and physical activity among older individuals (19). VR is a cost-effective and noninvasive approach for improving the lives of older adults in both clinical and recreational settings (20). Serious games (SG) are interactive, entertaining and engaging forms of exercise, providing therapeutic applications of VR for balance recovery and functional mobility (21). Evidence suggests VR and SG are an efficacious rehabilitation method of persons post-stroke and in persons with other neurological disorders, such as Multiple Sclerosis patients (22). SG can improve cognitive and physical functions on the basis of increased sensorial flow physical effort (23). Combining exercise with video games is promising for enhancing physical activity, social interaction, balance, and cognitive function among older adults. However, challenges include unfamiliar equipment, complex controls, and fast-paced displays. The use of VR by older people presents some potential risks, such as falls, fatigue or disorientation, which have been widely reported in the literature (24). Acceptability hinges on enjoyment and social interaction, while demand involves balancing physical and cognitive challenges with safety and technical considerations, with ongoing support and clear instructions being essential for sustained engagement (25).

Previous studies in literature have confirmed that VR represents a more experiential and effective method to develop and maintain a healthy lifestyle (26). The effect of VR gaming intervention on physical function and balance ability of older adults with impaired balance was better than that of conventional exercise or non-interventional training in some previous studies in the literature (27). There are still few studies that address the impact of virtual reality and serious games solutions on adherence to less sedentary lifestyles, on motivation to exercise, both in older adults living in nursing homes and in those who live independently at home.

Innovative interventions supported by IT can contribute to the care pathway only if innovative solutions are adequately integrated into care processes, professionals’ work routines and end-users’ daily lives. Knowledge of the care experience, held only by the patient, is particularly valuable. This knowledge can be enhanced through participatory design, in which the customer is no longer the passive recipient of a new product but is an integral part of the design and innovation process as a whole (28).

“3D Community Aware Virtual Spaces as Smart Living Environments for Physical Activity and Rehabilitation” (VR2Care) is an EU Horizon 2020 program funded project, aimed to develop and test VR solutions enabling multi-user experiences for physical activity and rehabilitation service provision for older adults (29, 30). The VR2Care project is coded with the active participation of older adults, caregivers, therapists, communities, and clinicians to provide a comprehensive single user and multi-user experience, leveraging digital technology.

**Figure 1:**
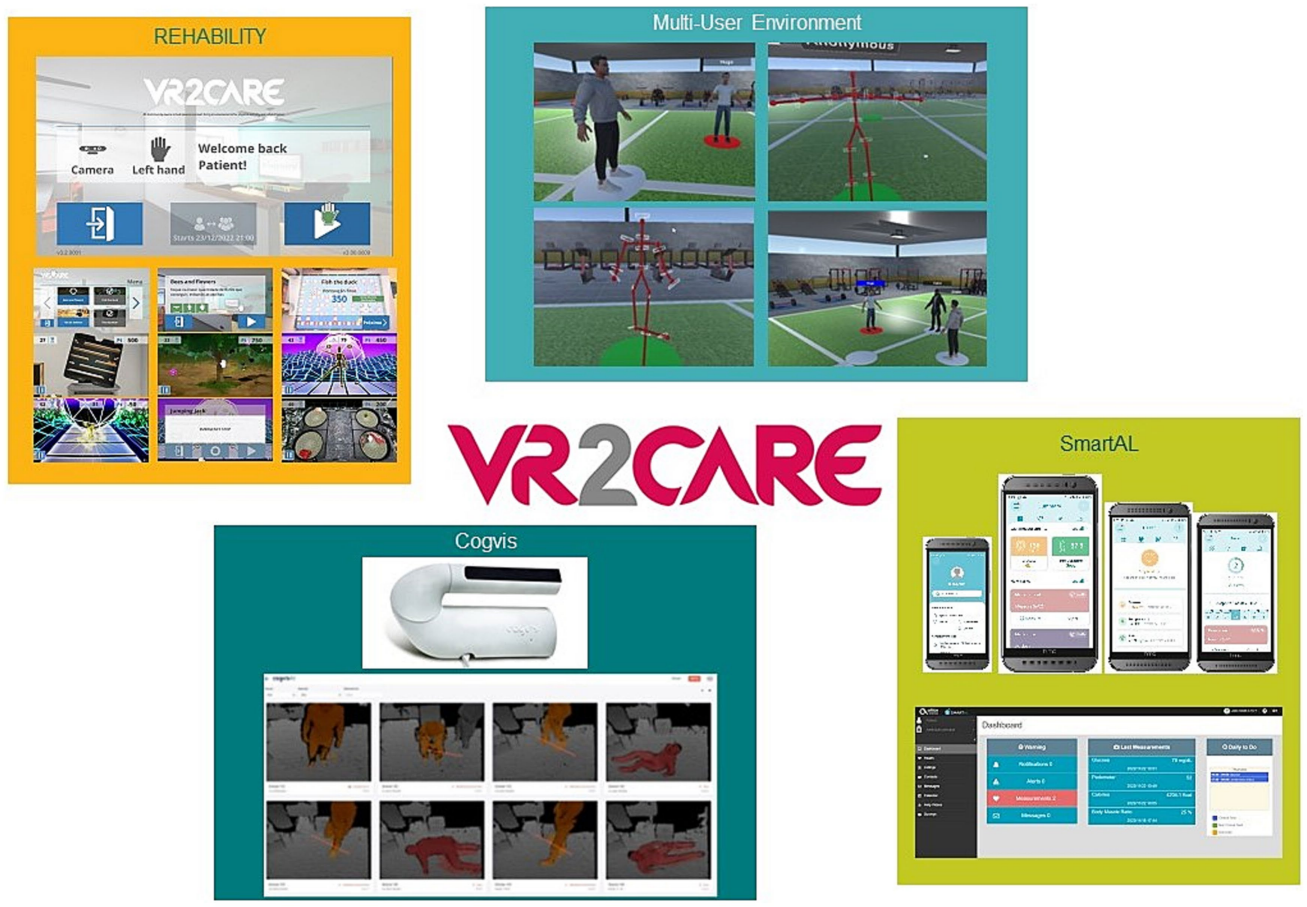
The four solutions integrated in the VR2Care system of systems.

The project consortium developed a system of systems (31) that combines 4 existing solutions (Figure 1), into a single integrated one:

- Rehability system combines physical with cognitive stimulation offering a range of serious games for functional exercises that patients can easily lead back to realistic tasks so that also older people and fragile patients understand the usefulness of what they are doing while enjoying the gamified setting (32).
- 3D Multiuser Environment (MUE) provides virtual environments for physical exercise that can be scheduled One-to-one, for private and more controlled physical exercise between the patient and the trainer, or One-to-many for groups to practice physical exercise with the support of a coach/trainer (33).
- SmartAL is a telemonitoring system that allows healthcare providers and patients to define and execute daily monitoring plans, including sending notifications for taking medications (34);
- cogvisAI is a Motion Capture and Metrics tool that detects falls, obstacles, movement and position while preserving patient privacy (35, 36).

As part of the project activities, four pilot sites from three European countries (Italy, Portugal, The Netherlands) explored the capabilities of the VR environment in a remote psychomotor training with exergames and a hybrid approach with local groups of older adults performing physical activities. The project has allowed us to develop a business model to promote physical activity in older adults, adopting the VR2CARE solution, integrated into the services of the pilot organizations. In the preparatory phase, the service path and the user scenario applied to older adults’ population who perform physical activity with VR2CARE solutions were identified.

The aim of the present study is to demonstrate how the use of the VR2CARE solution by older people improves physical activity and affects their perceived quality of life. In addition, the study aims to measure the level of satisfaction, usefulness and usability of the VR2CARE solution.

## 2 Materials and methods

### 2.1 Study design

**Figure 2:**
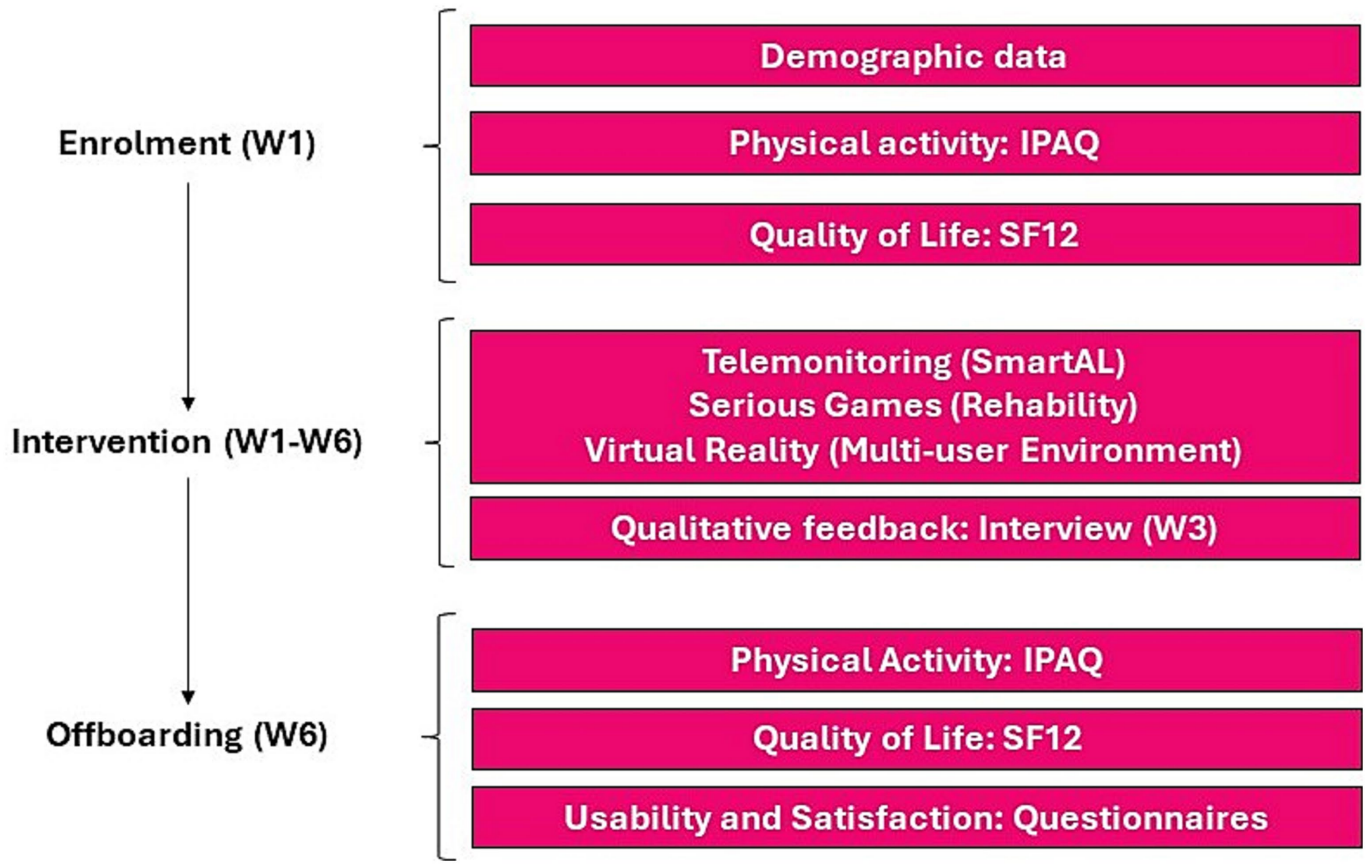
Pilot activities workflow.

The pilot study is a mixed method study, using qualitative and quantitative surveys to evaluate quality of life and physical activity of older users, and usability of the solution. The study is a quasi-experimental pre-post intervention with an only within-subject comparison. Data were collected at baseline and after a follow-up of 6 weeks. The data collection is a mix of investigator site data entry and users’ self-reported data through the solutions or through online and paper-based means. The indicators considered are the end-users self-reported outcomes via surveys, integrated into the solutions’ interfaces (quality of life, physical activity, usability, satisfaction and acceptance). The data collected and the means of measurement are reported in Table 1. During enrollment, personal data were collected (age, sex, marital status, and level of education) and data on the user’s self-perceived general health conditions (SF-12) and the level of physical activity performed (IPAQ-SF). During the offboarding phase, 6 weeks after enrolment, self-perceived general health conditions (SF-12) and the level of physical activity performed (IPAQ-SF) were administered again. In addition, during the offboarding, the level of acceptance, satisfaction and usability of the VR2Care solution by users were measured. Each phase of the pilot was implemented according to the scheme shown in Figure 2.

**Table 1:** Data collected and means of measurement.

Type of data	Description of data	Means of measurement
Age	Number of years	VR2Care Registration
Sex	Options: male, female	
Marital status	Options: single, married, widowed, divorced, in partnership	
Educational level	Options: Primary School or No Education, Secondary School, University Degree	
Quality of life	SF12 physical score SF12 mental score	SF12 Questionnaire
Physical activity	Intense activity MET* Moderate activity MET* Walking activity MET* Total MET*	IPAQ Questionnaire
Usability, satisfaction and acceptance	Satisfaction 1–5 score Usability 1–5 score Usefulness 1–5 score	Self-Assessment Questionnaire

The presented research is an exploratory investigation aimed at directing a subsequent randomized controlled trial (RCT) and long-term implementation (37). Data were collected during clinical practice, in accordance with Art. 89 of the General Data Protection Regulation, which allows the processing of personal data for archiving purposes in the public interest, scientific or historical research or statistical purposes, provided that technical and organizational measures are taken to guarantee the principle of data minimization (38). Baseline data from each patient served as referral to register eventual impact of the technology on registered clinical outcomes. Informed consent has been obtained from all subjects as per clinical practice. Statistical analysis was conducted on anonymized data. Only researchers involved in the activity of each participating center, have stored the personal data used for the research through the use of identification codes.

To ensure the security of the data processing of the study from the risks of abusive access or theft, file system encryption systems and the use of protected transmission channels were used. Since it is not a clinical study and the data are anonymized and processed in accordance with current data protection provisions, the protocol has not been submitted to the ethics committee for approval.

### 2.2 Settings

The pilot sites of the VR2Care project are organizations that, although they have different aims, provide adapted physical activity (APA) services for older adults in their own facilities.

#### 2.2.1 Federico II University and Hospital (UNINA)

UNINA provides in-hospital admittance, day hospitals, day services and outpatient activities. The Hospital hosts the Outpatient Clinic for the Prescription for APA for Patients with Chronic Diseases, which was launched in July 2019. This Clinic offers patients the possibility to follow prescriptions for APA that are tailored to their specific needs. The Clinic is open to patients from the hospital, as well as the GP and other hospitals of the territory. The VR2Care pilot activities were performed at UNINA, following a specialist medical visit, during which the clinician and physical trainers assessed physical parameters and prescribed a personalized exercise program, to be performed through the VR2Care solution. Once at home, using VR2Care, the patient accessed the prescript program.

#### 2.2.2 Cooperativa Sociale COOSS Marche ONLUS (COOSS)

COOSS is a non-profit organization providing social services to disadvantaged people throughout the Marche Region, Italy. COOSS manages a large number of facilities: residential facilities, daycare centers, family communities and nursing homes, mainly for older adults and disabled people. Some of them are managed on behalf of local authorities, while others are owned by COOSS. COOSS also provides territorial and home-based services, including care and home assistance to older adults and disabled people. COOSS focused primarily on users living in nursing homes and protected settings, where leisure, animation and physical exercise activities are proposed on a weekly basis. In a second phase of the VR2Care experimentation, users living alone in their own homes were involved. COOSS staff distributed the system in private homes and trained the formal caregiver, the family member/ informal caregiver and the user himself/herself in the use of VR2Care.

#### 2.2.3 TanteLouise

TanteLouise (TL) offers a complete package of services of home care, (assisted) living, healthcare, nursing and supplementary services in the municipalities of Bergen op Zoom, Woensdrecht and Steenbergen in the Netherlands. TanteLouise provides nursing care to over 1,150 clients in their care centers and nursing homes, daycare to more than 400 clients and specialist care in the home situation. TanteLouise has a track record of groundbreaking care for people with dementia. In addition to providing care, tanteLouise invests in innovative care concepts. The TanteLouise pilot activities focus on older adults living independently and receiving physiotherapy, first in the specialist rehabilitation center and later at home. TanteLouise selected two centers: a geriatric rehabilitation center (GRZ) and a nursing home (ABG). Users were trained to use the system under the supervision and support of a qualified physiotherapist. The physiotherapist installed the system in the users’ home and, when necessary, explained the use of VR2Care to a family member/informal caregiver. Depending on the physical training program, training was scheduled 1 or 2 times per week. Each session lasts from 15 to 30 min.

#### 2.2.4 Venerável Ordem Terceira de São Francisco do Porto (OSF)

OSF is a non-profit organization providing cultural, health and social services in the city of Porto, Portugal. OSF manages several facilities: two nursing homes for older adults, a UNESCO-listed cultural heritage site, health services, including a hospital, and a social canteen. OSF adopted the VR2Care system for therapies and physical activities within residences and nursing homes, allowing patients to have exclusive content to use under supervision, planned in advance. The OSF involved older adults living permanently in the nursing home, as well as people living in the independent house that exist at the facilities. The OSF physiotherapist identified users and APA programs to be carried out by them, through group sessions. The OSF doctors supervised all the health parameters and evaluate them. The older adults were accompanied by caregivers and professionals to get used to the system. Clinicians and physiotherapists monitored the progress in using the VR2CARE solution.

### 2.3 VR2Care participants

Participants were recruited from existing users at the pilot sites. Participation in the trial was strictly voluntary. The target trial user sample size was 15 per pilot site for a total of 60 users. Each participant was given the opportunity to use the VR2Care solution for 6 weeks. Enrollment took place in three cycles, with groups of 20 patients each (Table 2).

**Table 2:** Pilot evaluation phases.

Phase	Expected users per site	Duration	Solutions
Phase I	20 users	6 weeks	SmartAL Rehability
Phase II	20 users	6 weeks	SmartAL Rehability
Phase III	20 users	6 weeks	SmartAL Rehability MUE

Subjects with the following inclusion criteria were eligible to participate to the exploratory study: 65 + age; prescription for physical activity, APA by a specialist or recommendation for preventive physical training from the General Practitioner (GP); basic digital literacy; impossibility to attend a gym/therapy center for mobility or travel problems. Subjects were excluded if unable to consent or having an invalidating mental illness; compromised cognitive level; severe loss of independence; severe cardiovascular conditions.

Standardized data collection forms were adopted, which included continuous monitoring of data quality, to allow for the highest completion rate of the questionnaires.

### 2.4 Data collection

#### 2.4.1 Physical activity

The International Physical Activity Questionnaire Short Form (IPAQ-SF) (39) was used as a standardized measure to estimate habitual practice of physical activities based on four intensity levels: (1) vigorous-intensity activity, (2) moderate-intensity activity, (3) walking, and (4) sitting. The IPAQ-SF reliably measures change over time in physical activity in repeated measures studies (53). The questions contained in the tool are reported in Supplementary material 1. Two forms of output from scoring the IPAQ-SF were reported: as categories (low activity levels, moderate activity levels or high activity levels) and as metabolic equivalent of task (MET) minutes representing the amount of energy expended carrying out physical activity.

#### 2.4.2 Quality of life

The 12-item Short Form Survey (SF-12) (40) questionnaire allowed statements about the user’s state of health. The SF-12 version adopted in VR2Care is non-proprietary and the questions contained in the tool are reported in Supplementary material 2. Two summary scores are reported from the SF-12—a mental component score (MCS-12) and a physical component score (PCS-12).

#### 2.4.3 Usability, satisfaction and acceptance

A 12-questions survey was administered post-intervention to users for the assessment of satisfaction, self-management and usability (34). In order to build the questionnaire, a 5-point Likert scale was used as a psychometric scale to assess users’ opinions regarding VR2Care solution (41, 42) for the physical activity and rehabilitation. The questionnaire was based on the Questionnaire for User Interaction Satisfaction (QUIS7) instrument (43). The questions contained in the tool are reported in Supplementary material 3.

#### 2.4.4 Statistical analysis

Data are expressed as mean ± standard deviation (SD) unless otherwise stated. Within the group, baseline to end of observation differences were assessed by paired sample *t*-test. Statistical significance was set at *p* < 0.05. Cohen’s d conventional effect size cutoffs have been adopted [0.2 (small effect), 0.5 (moderate effect), and 0.8 (large effect)]. The statistical analysis was performed according to standard methods using the Statistical Package for Social Science (SPSS) software V.28 (SPSS/PC).

## 3 Results

### 3.1 Demographic data

A total of *n* = 75 users were enrolled in the study, distributed across the VR2Care pilot sites. The average age of the population is 79.75 (±9.96) and they are almost equally distributed into age classes. The youngest enrolled user is 65 years old. The oldest enrolled user is 95 years old. 62.6% (*n* = 47) of users are women. 70.6% (*n* = 53) of users are single, divorced, or widowed. 44% (*n* = 33) of users have a low level of education (Table 3).

**Table 3:** Participants demographic data (*n* = 75).

Characteristic	Value
Age (years), mean (SD)	79.75 (9.96)
Gender, female, n (%)	47 (62.6)
Minimum age in years	65
Maximum age in years	95
Age classes, n (%)	
≤75	23 (30.6)
76–84	23 (30.6)
≥85	29 (38.6)
Marital status, n (%)	
Single	13 (17.3)
Married	22 (29.3)
Widowed	35 (46.6)
Divorced	5 (6.6)
Level of education, n (%)	
Primary school	33 (44)
Secondary school	28 (37.3)
University degree	14 (18.7)
User per pilot site, n (%)	
COOSS	20 (26.7)
UNINA	10 (13.3)
TL	15 (20)
OSF	30 (40)

### 3.2 Physical activity

No significant improvement in physical activity was found (Table 4). Little improvement, although not significant, in physical activity was found, comparing the Total MET average value of users who participated in phase I and II, therefore using SmartAL and Rehability. No significant improvement in physical activity was found, comparing the Total MET average value of users ≤75. Little improvement, although not significant, in physical activity applies in ≥76 population.

**Table 4:** MET comparison at baseline and after 6 weeks intervention (*N* = 75).

	Baseline	After 6 weeks intervention		
Variables	Mean (SD)	Mean (SD)	*P-*value	Cohen’s *d*
Intense activity MET	172.80 (574.49)	190.27 (573.79)	0.31	0.03
Moderate activity MET	287.47 (772.71)	301.87 (699.32)	0.53	0.01
Walking activity MET	807.00 (914.99)	805.80 (879.42)	0.97	<0.01
Total MET	1279.27 (1237.00)	1307.40 (1177.35)	0.49	0.02
Phases				
Phases I-II (*N* = 49)				
Intense activity MET	205.71 (645.19)	243.33 (666.46)	0.10	0.06
Moderate activity MET	362.45 (919.94)	367.35 (823.55)	0.88	0.01
Walking activity MET	607.35 (749.01)	615.92 (743.60)	0.64	0.01
Total MET	1197.55 (1292.61)	1243.67 (1233.65)	0.28	0.04
Phase III (*N* = 26)				
Intense activity MET	110.77 (414.20)	92.31 (333.09)	0.33	0.05
Moderate activity MET	146.15 (332.73)	178.46 (348.02)	0.20	0.09
Walking activity MET	1183.27 (1084.26)	1163.65 (1011.80)	0.82	0.02
Total MET	1433.27 (1132.96)	1427.50 (1076.11)	0.95	0.01
Age classes				
≤75 age (*N* = 23)				
Intense activity MET	473.04 (956.02)	466.08 (933.77)	0.82	0.01
Moderate activity MET	401.73 (667.74)	406.95 (675.67)	0.79	0.01
Walking activity MET	751.30 (975.50)	713.47 (852.91)	0.71	0.04
Total MET	1618.26 (1398.65)	1578.69 (1316.67)	0.68	0.03
76–84 age (*N* = 23)				
Intense activity MET	62.60 (219.69)	104.34 (248.84)	0.16	0.18
Moderate activity MET	146.08 (245.27)	198.26 (364.71)	0.33	0.17
Walking activity MET	656.73 (664.32)	655.43 (641.09)	0.96	<0.01
Total MET	886.30 (711.29)	978.91 (765.17)	0.19	0.13
≥85 age (*N* = 29)				
Intense activity MET	22.06 (92.94)	34.28 (181.42)	0.54	0.08
Moderate activity MET	308.96 (1.073.67)	300.68 (900.76)	0.84	0.01
Walking activity MET	970.34 (1.034.14)	998.27 (1.041.87)	0.10	0.03
Total MET	1322.06 (1373.45)	1352.75 (1299.99)	0.57	0.02

Regarding physical activity, at baseline, 57.3% (*n* = 43) of users are sufficiently or very active. At follow up, 9% of users improved physical activity from Inactive to Sufficiently Active (Table 5).

**Table 5:** Distribution of users by physical activity level (*N* = 75).

	Baseline	After 6 weeks intervention
Inactive (MET <700), n (%)	32 (42.7)	29 (38.7)
Sufficiently active (MET 700–2,519), n (%)	31 (41.3)	34 (45.3)
Active or very active (MET ≥2,520), n (%)	12 (16)	12 (16)

### 3.3 Quality of life

As shown in Table 6, on average, users scored better for the mental component than for the physical component. No significant improvement in quality of life was found. The same applies if we compare the SF-12 average value of users who participated in phase I and II, therefore using SmartAL and Rehability, and those who participated in phase III, using SmartAL, Rehability and MUE. No significant improvement in quality of life was found comparing the SF-12 average value of users according to age class.

**Table 6:** Quality of life comparison at baseline and after 6 weeks intervention (*N* = 75).

	Baseline	After 6 weeks intervention		
Variables	Mean (SD)	Mean (SD)	*P*-value	Cohen’s *d*
PCS-12 (physical score)	44,69766 (8.57)	44,89967 (8.84)	0.56	0.02
MCS-12 (mental score)	51,61674 (10.23)	50,75724 (10.45)	0.08	0.08
Phases				
Phases I-II (*n* = 49)				
PCS-12 (physical score)	44.49115 (8.38)	44.92787 (8.83)	0.39	0.05
MCS-12 (mental score)	52.74932 (8.97)	51.56392 (9.40)	0.10	0.13
Phase III (*n* = 26)				
PCS-12 (physical score)	45.08685 (9.07)	44.84653 (9.03)	0.42	0.03
MCS-12 (mental score)	49.48227 (12.17)	49.23695 (12.24)	0.58	0.02
Age classes				
≤75 age (*n* = 23)				
PCS-12 (physical score)	46.87380 (7.60)	47.55067 (8.04)	0.22	0.09
MCS-12 (mental score)	51.40157 (10.47)	50.50070 (10.75)	0.25	0.08
76–84 age (*n* = 23)				
PCS-12 (physical score)	42.63888 (8.64)	42.60082 (8.91)	0.96	<0.01
MCS-12 (mental score)	50.72369 (11.39)	49.92659 (11.99)	0.55	0.07
≥85 age (*n* = 29)				
PCS-12 (physical score)	44.60458 (9.08)	44.62039 (9.12)	0.97	<0.01
MCS-12 (mental score)	52.49567 (9.32)	51.61948 (9.13)	0.04	0.09

### 3.4 Usability, satisfaction, and acceptance

**Figure 3:**
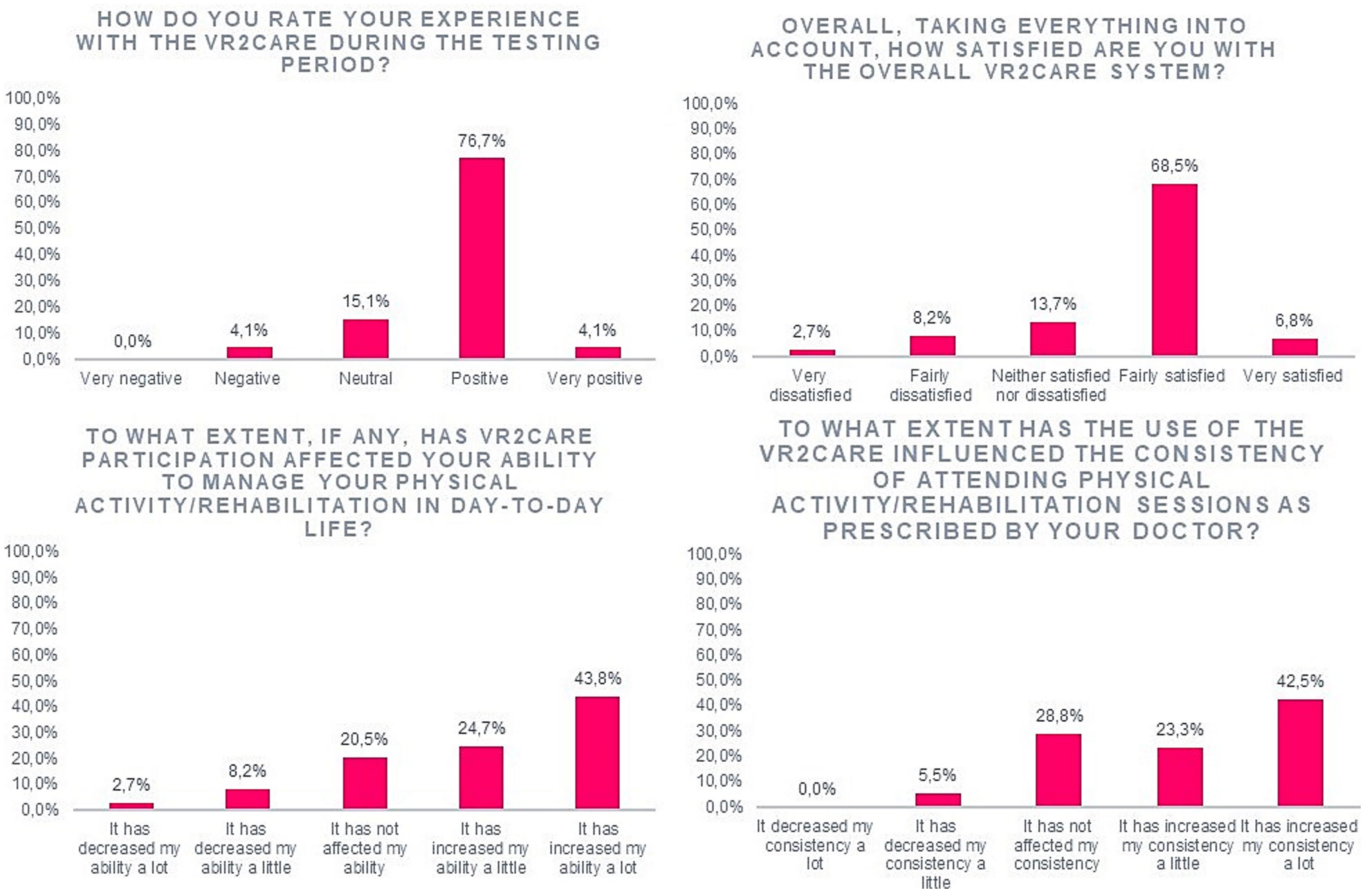
Results on users’ satisfaction.

The experience with the VR2Care during the testing period is considered positive by 76.7% of the patients and 4.1% of them considered that it as was very positive. Most users interviewed were fairly satisfied (68.5%) with VR2Care. The results regarding the way that the participation in the pilot affected the user’s ability to manage their condition on a day-to-day basis were also very encouraging: 24.7% of the users considered that by participating in the study they increased a little bit their ability to manage their physical activity; and 43.8% considered that that by participating in the study they increased a lot their ability to manage physical activity. VR2Care increased the consistency of attending physical activity sessions prescribed by the doctor in 65.8% of users (Figure 3).

Using the VR2Care system, 72.6% of the patients considered that effort is mostly worth it and, more importantly, 9.6% of the patients considered that effort as totally worth it.

**Figure 4:**
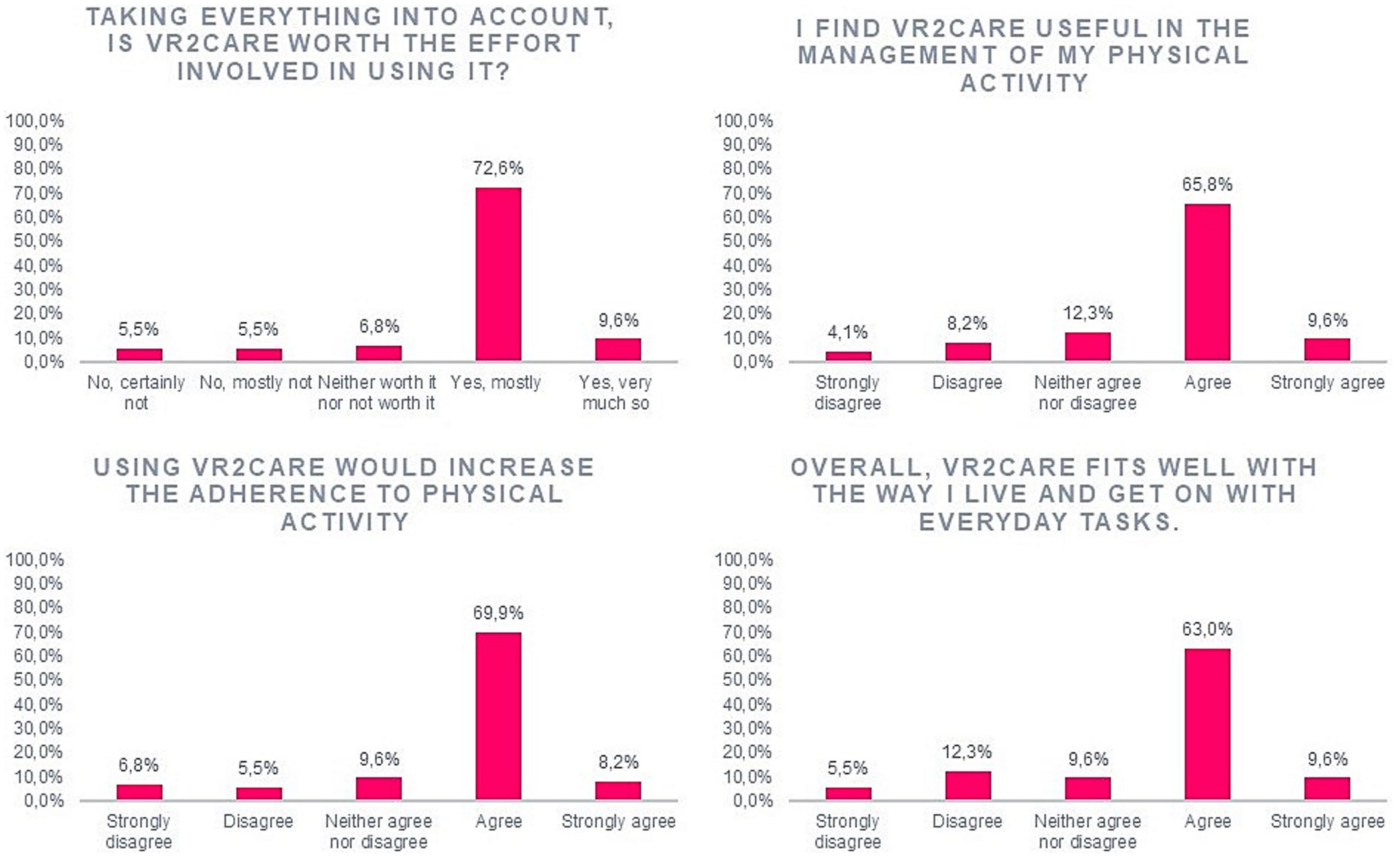
Results on perceived usefulness.

When questioned about the usefulness of the VR2Care system in the management of their physical activity, 65.8% of the users agreed that it is useful while 9.6% of the users strongly agreed that it is useful. The users agreed with the fact that the VR2Care system increased adherence to physical activity, 65.8% of them agreed and 9.6% strongly agreed with that sentence. The patients agreed with the fact that the VR2Care system fitted with their way of living (63.0%) (Figure 4).

**Figure 5:**
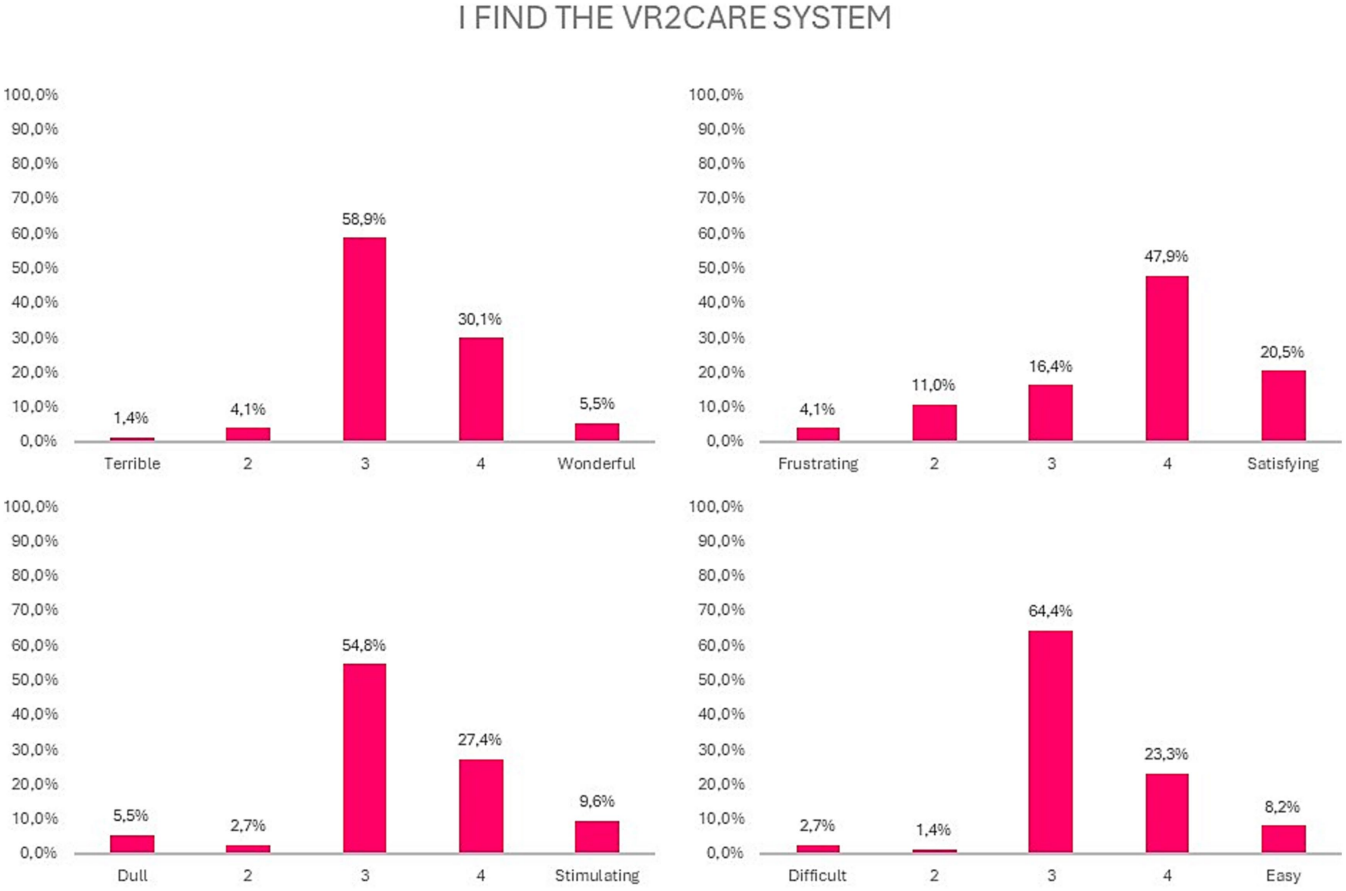
Results on usability of the VR2Care solution.

Regarding the way that the users perceive the VR2Care system: only 5.5% considered it as being wonderful; 20.5% considered it as being satisfying; 9.6% considered it as being stimulating; more than 8.2% considered it as being easy (Figure 5).

**Figure 6:**
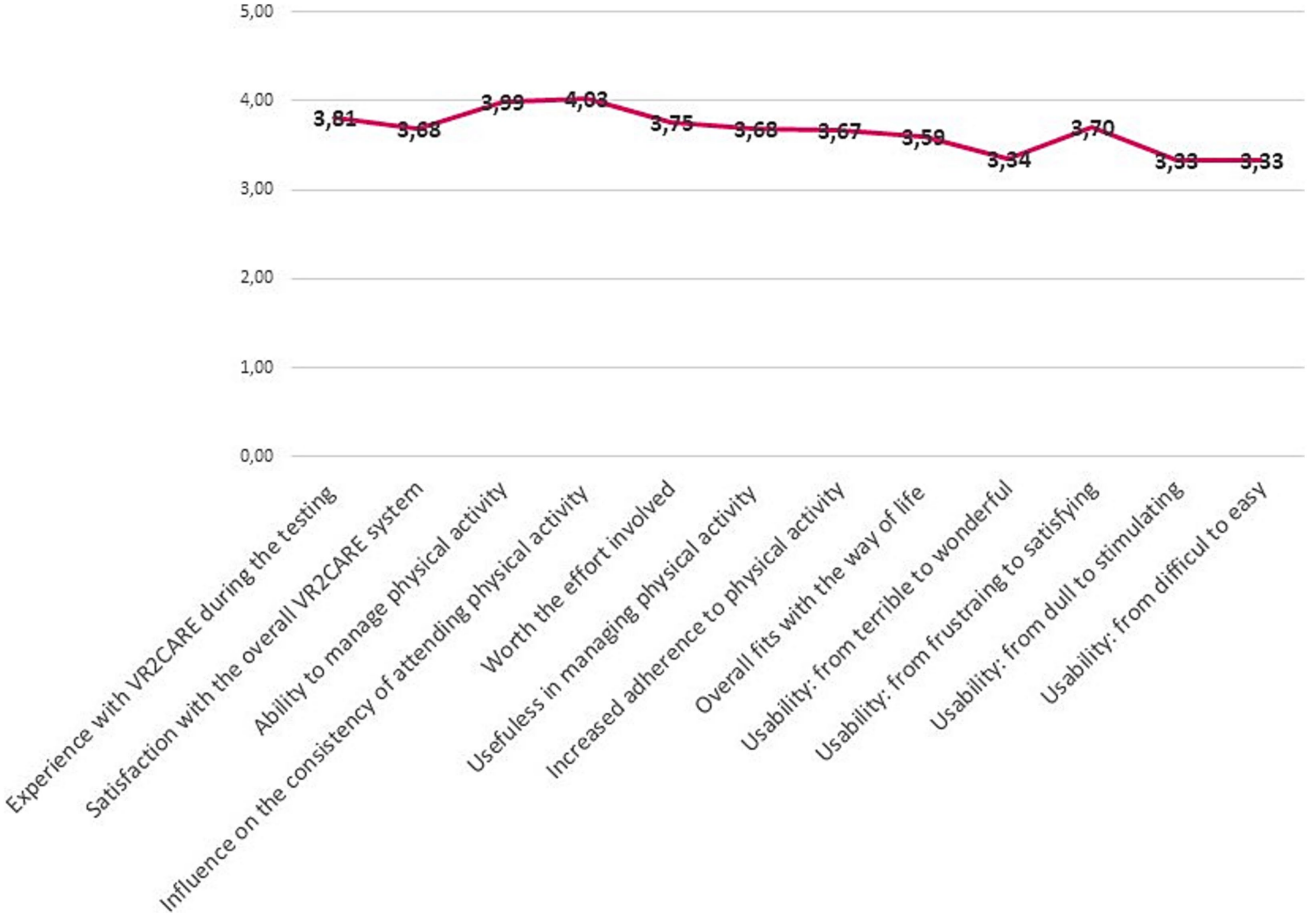
Overall satisfaction, usefulness and usability results.

The collected data regarding each question is generally positive. VR2Care was appreciated mostly for its usefulness in managing physical activity and the capacity to influence the consistency of attending physical activity sessions as prescribed by doctor (Figure 6).

**Figure 7:**
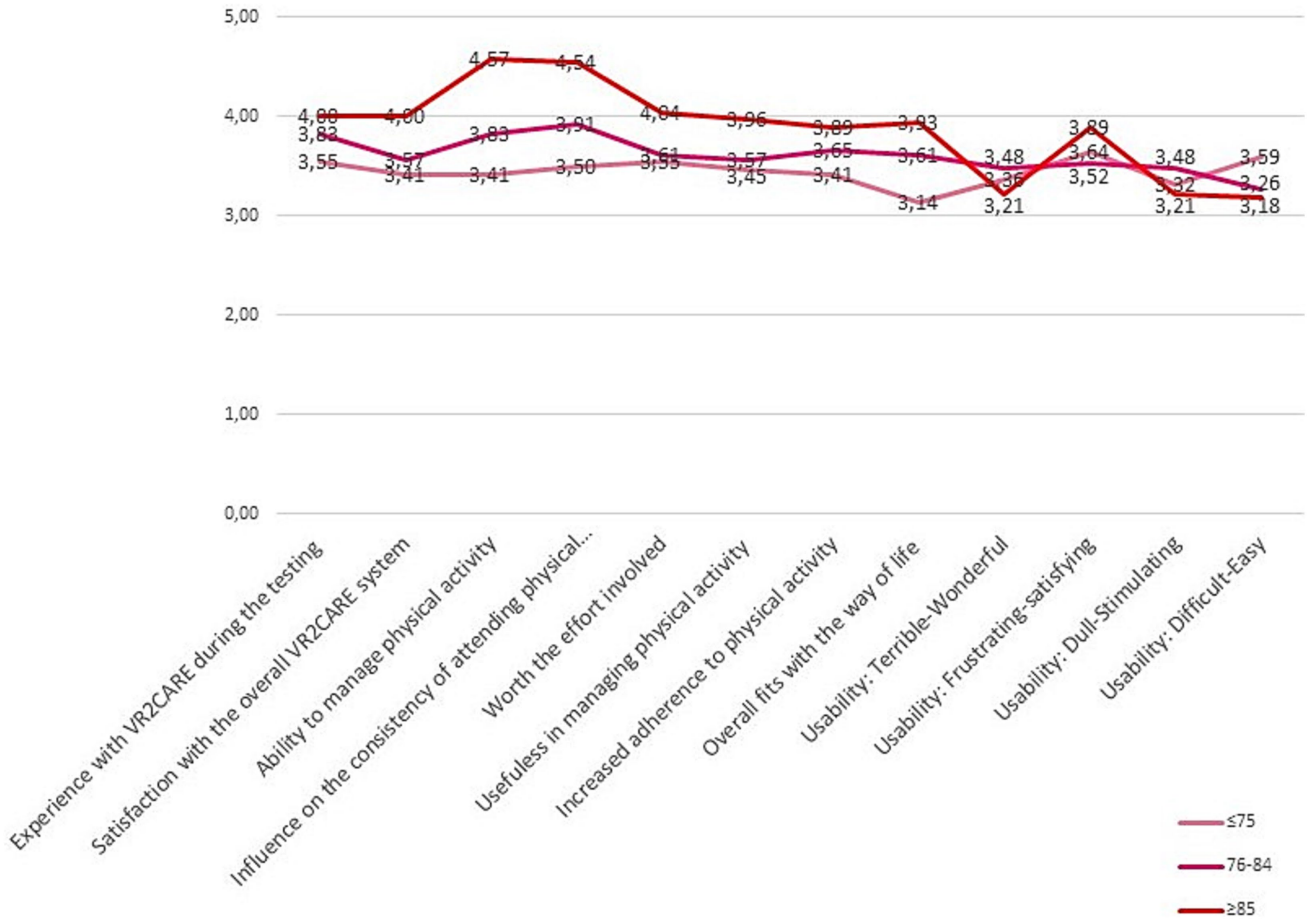
Satisfaction, usefulness and usability results per age class.

The solution was most appreciated in adults over 85 years old (Figure 7).

**Figure 8:**
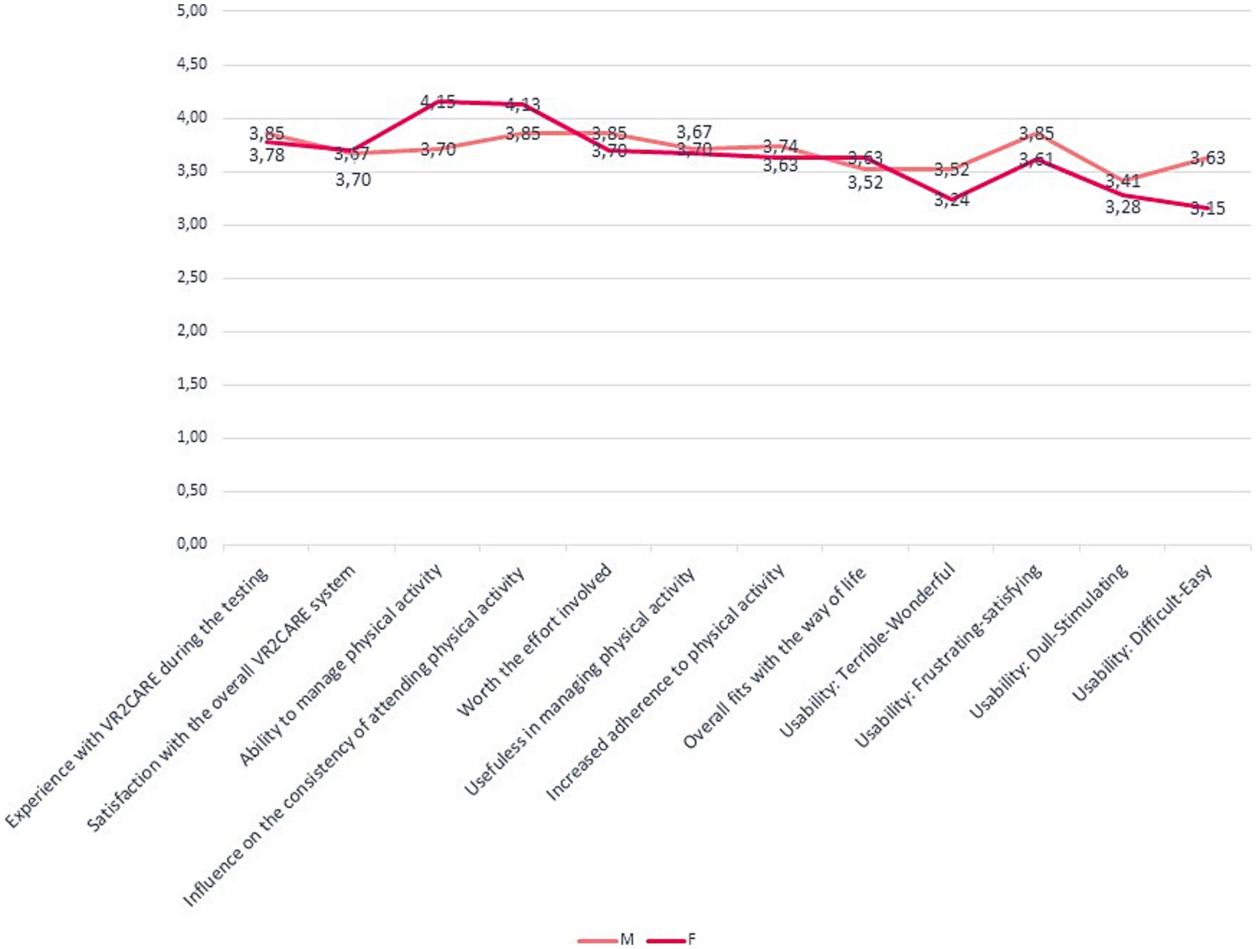
Satisfaction, usefulness and usability results per gender.

Women appreciate VR2Care’s usefulness in managing physical activity and its ability to influence the consistency of participation in physical activity sessions more, but express themselves less positively regarding the usability of the solution (Figure 8).

**Figure 9:**
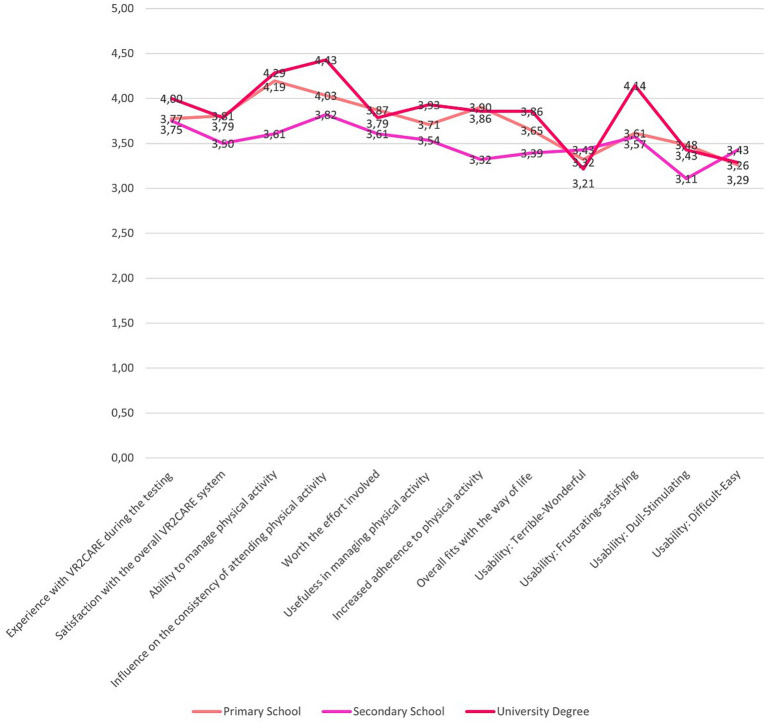
Satisfaction, usefulness and usability results per level of education.

The level of education positively influences the usability and acceptance of VR2Care (Figure 9).

## 4 Discussion

Digital transformation in the health and care sector requires appropriate tools that offer decision makers sufficient knowledge of the potential, benefits and costs associated with the introduction and use of innovative IT solutions (44).

The present study reports the results of the exploratory trial of VR2Care, a digital solution for physical activity and rehabilitation supported by SG and VR. The study aimed to explore and measure the level of user compliance, effectiveness, satisfaction and impact on the quality of life and physical activity of older adults.

No significant improvement was found in the quality of life and physical activity of users over the 6-week observation period. The short duration of the intervention (6 weeks) might not allow for significant changes in the lifestyle of users regarding physical activity and quality of life. Lifestyle changes require coordinated and long-term interventions, capable of modifying the users’ daily habits. The average age of the enrolled population is high (79.76). The older the age, the lower the life expectancy and consequently the self-perceived quality of life does not increase. Future studies with a rigorous design, such as measuring steps and heart rate in a larger sample, and more follow-up assessments over a longer period, are needed. As previous studies have shown, SG and VR intervention has been shown to be a promising strategy to improve balance control and reduce falls in older adults (26, 45), but there is no significant difference in the improvement of physical and cognitive function between exergaming and conventional exercise (46). Evidence on whether SG and VR improve exercise adherence and less sedentary lifestyles in older adults is still scarce.

Previous studies have shown that if older people have minimal interaction with technology on a daily basis, it may hinder their interest in new technologies and subsequent browsing experiences. In the early phase of adoption of a new technology by older adults with a low level of computer literacy (IT), a lot of support is needed from operators and professionals who take care of the users (47). The support of an operator or a caregiver present at every session was not always possible during the VR2Care pilot, especially in those organizations that provided the service at the user’s home. Users who participated in phase III and those over 85 years old were those who received more support from operators and carers and for them the results are slightly better. The level of maturity of the MUE solution does not yet allow for training sessions to be carried out independently or in the classroom, remotely. There are still many technical issues that need to be addressed to make the use of the solution more independent from operators. Further development of the solution, especially MUE, is necessary to allow greater accessibility by older adult patients and replace services in the presence of an operator/carer.

The present study reports survey results on satisfaction, usability and usefulness of the VR2Care tool. User opinions are generally positive. VR2Care was appreciated above all for its usefulness in managing physical activity and for its ability to influence the consistency of participation in physical activity sessions prescribed by the doctor. Users are satisfied with using the proposed solution for physical activity and perceive the usefulness of VR2Care, because probably they appreciated the opportunity to exercise through VR2Care but at the same time they perceive its low maturity and the difficulties in using the solution independently.

### 4.1 Barriers and practical implications

Despite the study did not having a significant impact on physical activity and quality of life, it obtained positive results in user satisfaction. The present study, in some pilot sites (COOSS and OSF) was conditioned by the presence of operators during physical exercise sessions, in others on the contrary by the absence of operators and difficulty in using the solution independently (UNINA and TL).

IT allow for the provision of care at reduced costs, but specific and objective methods must be developed to evaluate the clinical quality of new technologies and to definitively demonstrate the advantages of VR, AR, gamification and telerehabilitation compared to conventional face-to-face exercise (48). The results of the exploratory study demonstrate that it is necessary to measure the effects of the use of VR and SG solutions in community centers and in tele-rehabilitation at the user’s home to better understand which approach is more effective.

The low level of IT literacy of the users involved might impact the results of the exploratory study and the motivation to use the VR2Care solution for physical exercise and telemonitoring. The intervention was not only aimed at users with a minimum level of IT Literacy, but the exploratory trial aimed to verify whether the solution could be adopted, in contexts of use, such as nursing homes, where the support of the operators could allow the user to carry out the exercises, even if they do not use IT.

As well as, the exploratory trial allowed to map the key players and critical points, both technical and organizational, for the adoption of IT solutions to improve physical activity and lifestyle. Results from exploratory trial will be pivotal to implement a randomized clinical trial, to measure the impact of the digital intervention on adherence to physical activity and on health outcomes in target population, such as chronic non-communicable diseases patients. The results of the exploratory study indicate that an important aspect to evaluate in a future RCT is the effectiveness of VR-supported interventions on improving physical and cognitive functions in older adults with mild or severe cognitive decline.

The adoption of innovative solutions varies between different countries (49) so it is necessary to develop effective business models to implement and adopt them (50). IT can only contribute to the care path if the innovative solutions are adequately integrated into the assistance processes, work routines and daily life of end users.

Since the organizations involved in the pilot have different goals and different expectations in using the VR2Care solution, further studies should develop organizational models dedicated to the specific needs of the users that they want to address (rehabilitation, lifestyle, socialization) to order to avoid that the nature of the organization where participants are recruited could affect individual experiences.

Knowledge of the care experience, held only by the user, is particularly valuable. This knowledge can be enhanced through participatory design, in which the customer is no longer the passive recipient of a new product but is an integral part of the design and of the innovation process as a whole (51).

The progressive integration of digital technologies in diagnostic-therapeutic pathways represents a good starting point and is driving innovative educational and training paths that promise to contribute to opening new opportunities for sustainable development. There is still a long way to go, especially in relation to the use of VR to promote healthy lifestyles, physical activity and rehabilitation to enable personalized interventions, and the environmental elements that hinder the adoption of this type of solutions at the user’s home.

### 4.2 Limitations

Exploratory study has several limitations that should be addressed in future studies. The small sample size and the length of follow-up affected the results. The lack of a control group, the heterogeneity of the sample and the limited duration of follow-up represent important limitations of the exploratory study. Future randomized controlled trials should focus on a more heterogeneous population and include a control group receiving conventional exercise programs to compare with innovative services. In the future, it would be appropriate to plan a longer enrollment period and a follow-up of at least 6 months to be able to record improvements in adherence to physical activity. The lack of collaboration with primary care physicians did not allow to monitor the clinical parameters of patients and to integrate the digital intervention with the care services of specific pathologies. In the future, it would be appropriate to implement a randomized clinical trial, for specific patient targets, such as chronic non-communicable diseases, to measure the impact of the digital intervention on adherence to physical activity and on health outcomes. During the enrolment, information on the percentage of potential ineligible participants and their characteristics was not collected. This type of information could be very useful to identify the target population in further future studies. In all pilot sites, difficulties were encountered in implementing the pilot due to technical problems that did not allow users to use the solution independently. A further problem encountered by users is the low level of maturity of the MUE solution which did not allow users to use the solution through collective sessions and with the virtual presence of a professional. The installation of VR2Care at the user’s home which presupposes certain environmental conditions to be able to carry out the exercises, such as: free environment in front of the TV (no furniture); have appropriate lighting conditions; put the camera at the right height and position; wear appropriate clothes. The absence of these conditions resulted in the inability of users to use the solution at home and the frustration in not being able to resolve problems without the support of an operator/caregiver.

## 5 Conclusion

The participants who used the VR2Care solution gave a favorable opinion on using them, stating that both created a positive experience during the test phase and increased their ability to manage physical activity. In an increasing burden scenario for health systems, SG and VR solutions can provide a similar level of healthcare services for the management of physical activity and rehabilitation, with reduced costs and saving time for operators and patients. Our results are encouraging and suggest that randomized controlled trials are needed to assess correlations between specific features of the solution and health outcomes. The study suggests addressing VR and SG services for physical activity to a more heterogeneous sample and measuring adherence through more valid tools. Digital technologies for older adults must address the problem of lack of IT literacy to provide explicit information on the use of solutions, potential health benefits including educational content, reminders and feedback.

## Data Availability

The raw data supporting the conclusions of this article will be made available by the authors, without undue reservation.
