# Supplementary material for: Virtual reality solution to promote adapted physical activity in older adults: outcomes from VR2Care project exploratory study
Source: Front Public Health. 2025 May 13;13:1584406. doi: 10.3389/fpubh.2025.1584406 (PMC12106364; doi:10.3389/fpubh.2025.1584406)
Supplement: Supplementary file 1 [file Data_Sheet_1.docx]

| IPAQ | | |
| --- | --- | --- |
| 1 | During the last 7 days, on how many days did you do vigorous physical activities like heavy lifting, digging, aerobics, or fast bicycling? | n. days per week ____  No vigorous physical activities so Skip to question 3 |
| 2 | How much time did you usually spend doing vigorous physical activities on one of those days? | n. hours per day ____  minutes per day ____  Don’t know/Not sure |
| 3 | During the last 7 days, on how many days did you do moderate physical activities like carrying light loads, bicycling at a regular pace, or doubles tennis? Do not include walking. | n. days per week ____  No moderate physical activities so Skip to question 5 |
| 4 | How much time did you usually spend doing moderate physical activities on one of those days? | n. hours per day ____  n. minutes per day ____  Don’t know/Not sure |
| 5 | During the last 7 days, on how many days did you walk for at least 10 minutes at a time? | n. days per week ____  No walking so Skip to question 7 |
| 6 | How much time did you usually spend walking on one of those days? | n. hours per day ____  n. minutes per day ____  Don’t know/Not sure |
| 7 | During the last 7 days, how much time did you spend sitting on a week day | n. hours per day ____  n. minutes per day ____  Don’t know/Not sure |
