# Supplementary material for: Virtual reality solution to promote adapted physical activity in older adults: outcomes from VR2Care project exploratory study
Source: Front Public Health. 2025 May 13;13:1584406. doi: 10.3389/fpubh.2025.1584406 (PMC12106364; doi:10.3389/fpubh.2025.1584406)
Supplement: Supplementary file 2 [file Data_Sheet_2.docx]

| SF12 | | |
| --- | --- | --- |
| 1 | In general, would you say your health is | - Excellent - Very good - Good - Fair - Poor |
| The following questions are about activities you might do during a typical day. Does your health now limit you in these activities? If so, how much? | | |
| 2 | Moderate activities such as moving a table, pushing a vacuum cleaner, bowling, or playing golf | - YES, limited a lot - YES, limited a little - NO, not limited at all |
| 3 | Climbing several flights of stairs. | - YES, limited a lot - YES, limited a little - NO, not limited at all |
| During the past 4 weeks, have you had any of the following problems with your work or other regular daily activities as a result of your physical health? | | |
| 4 | Accomplished less than you would like. | - Yes - No |
| 5 | Were limited in the kind of work or other activities | - Yes - No |
| During the past 4 weeks, have you had any of the following problems with your work or other regular daily activities as a result of any emotional problems (such as feeling depressed or anxious)? | | |
| 6 | Accomplished less than you would like | - Yes - No |
| 7 | Did work or activities less carefully than usual | - Yes - No |
| 8 | During the past 4 weeks, how much did pain interfere with your normal work (including work outside the home and housework)? | - Not at all - A little bit - Moderately - Quite a bit - Extremely |
| These questions are about how you have been feeling during the past 4 weeks. For each question, please give the one answer that comes closest to the way you have been feeling. How much of the time during the past 4 weeks… | | |
| 9 | Have you felt calm & peaceful? | - All of the time - Most of the time - A good bit of the time - Some of the time - A little of the time - None of the time |
| 10 | Did you have a lot of energy? | - All of the time - Most of the time - A good bit of the time - Some of the time - A little of the time - None of the time |
| 11 | Have you felt down-hearted and blue? | - All of the time - Most of the time - A good bit of the time - Some of the time - A little of the time - None of the time |
| 12 | During the past 4 weeks, how much of the time has your physical health or emotional problems interfered with your social activities (like visiting friends, relatives, etc.)? | - All of the time - Most of the time - Some of the time - A little of the time - None of the time |
