# Supplementary material for: Virtual reality solution to promote adapted physical activity in older adults: outcomes from VR2Care project exploratory study
Source: Front Public Health. 2025 May 13;13:1584406. doi: 10.3389/fpubh.2025.1584406 (PMC12106364; doi:10.3389/fpubh.2025.1584406)
Supplement: Supplementary file 3 [file Data_Sheet_3.docx]

| Usability, satisfaction and acceptance questionnaire | | |
| --- | --- | --- |
| 1 | How do you rate your experience with the VR2CARE during the testing period? | - Very negative (1) - Negative (2) - Neutral (3) - Positive (4) - Very positive (5) |
| 2 | Overall, taking everything into account, how satisfied are you with the overall VR2CARE system? | - Very dissatisfied (1) - Fairly dissatisfied (2) - Neither satisfied nor dissatisfied (3) - Fairly satisfied (4) - Very satisfied (5) |
| 3 | To what extent, if any, has VR2CARE participation affected your ability to manage your physical activity/rehabilitation in day-to-day life? | - It has decreased my ability a lot (1) - It has decreased my ability a little (2) - It has not affected my ability (3) - It has increased my ability a little (4) - It has increased my ability a lot (5) |
| 4 | To what extent, if any, has the use of the VR2CARE influenced the consistency of attending physical activity/rehabilitation sessions as prescribed by your doctor? | - It decreased my consistency a lot (1) - It has decreased my consistency a little (2) - It has not affected my consistency (3) - It has increased my consistency a little (4) - It has increased my consistency a lot (5) |
| 5 | Again, taking everything into account, is VR2CARE worth the effort involved in using it? | - No, certainly not (1) - No, mostly not (2) - Neither worth it nor not worth it (3) - Yes, mostly (4) - Yes, very much so (5) |
| 6 | To what extent do you agree with the following statements: | |
|  | I find VR2CARE useful in the management of my physical activity/rehabilitation. | - Strongly disagree (1) - Disagree (2) - Neither agree nor disagree (3) - Agree (4) - Strongly agree (5) |
|  | Using VR2CARE would increase the adherence to physical activity/rehabilitation. | - Strongly disagree (1) - Disagree (2) - Neither agree nor disagree (3) - Agree (4) - Strongly agree (5) |
|  | Overall, VR2CARE fits well with the way I live and get on with everyday tasks. | - Strongly disagree (1) - Disagree (2) - Neither agree nor disagree (3) - Agree (4) - Strongly agree (5) |
| 7 | I find the VR2CARE system | |
|  | Terrible-Wonderful | 1-5 |
|  | Frustrating-satisfying | 1-5 |
|  | Dull-Stimulating | 1-5 |
|  | Difficult-Easy | 1-5 |
